# Fetuin-A Deficiency but Not Pentraxin 3, FGF-21, or Irisin, Predisposes to More Serious COVID-19 Course

**DOI:** 10.3390/biom11101422

**Published:** 2021-09-28

**Authors:** Michał Kukla, Tomasz Menżyk, Marcin Dembiński, Marek Winiarski, Aleksander Garlicki, Monika Bociąga-Jasik, Magdalena Skonieczna, Dorota Hudy, Barbara Maziarz, Beata Kuśnierz-Cabala, Maria Kapusta, Lubomir Skladany, Ivica Grgurevic, Ivana Mikolasevic, Tajana Filipec-Kanizaj, Małgorzata Wójcik-Bugajska, Tomasz Grodzicki, Tomasz Rogula, Dominika Stygar

**Affiliations:** 1 Department of Internal Medicine and Geriatrics, Faculty of Medicine, Jagiellonian University Medical College, Jakubowskiego 2, 30-688 Kraków, Poland; michal.kukla@uj.edu.pl (M.K.); mwojcik@su.krakow.pl (M.W.-B.); tomasz.grodzicki@uj.edu.pl (T.G.); 2 Department of Endoscopy, University Hospital in Kraków, Jakubowskiego 2, 30-688 Kraków, Poland; marcin.dembinski@uj.edu.pl (M.D.); mwiniar@mp.pl (M.W.); 3 Department of Internal Medicine, Gastroenterology and Acute Intoxication, Regional Hospital, Lwowska 178A, 33-100 Tarnów, Poland; tomasz.menzyk@gmail.com; 4 2nd Department of General Surgery, Faculty of Medicine, Jagiellonian University Medical College, Jakubowskiego 2, 30-688 Kraków, Poland; 5 Chair of Gastroenterology, Hepatology and Infectious Diseases, Faculty of Medicine, Jagiellonian University Medical College, Jakubowskiego 2, 30-688 Kraków, Poland; aleksander.garlicki@uj.edu.pl (A.G.); monika.bociaga-jasik@uj.edu.pl (M.B.-J.); 6 Department of Systems Biology and Engineering, Silesian University of Technology, Akademicka 16, 44-100 Gliwice, Poland; magdalena.skonieczna@polsl.pl (M.S.); dorota@hudy.pl (D.H.); 7 Biotechnology Centre, Silesian University of Technology, Krzywoustego 8, 44-100 Gliwice, Poland; 8 Department of Diagnostics, Chair of Clinical Biochemistry, Faculty of Medicine, Jagiellonian University Medical College, Jakubowskiego 2, 30-688 Kraków, Poland; mbmaziar@cyf-kr.edu.pl (B.M.); beata.kusnierz-cabala@uj.edu.pl (B.K.-C.); maria.kapusta@uj.edu.pl (M.K.); 9 Department of Internal Medicine and HEGITO (Hepatology, Gastroenterology and Liver Transplantation), F.D. Roosevelt University Hospital, Nam. l. Svobodu 1, 975 17 Banska Bystrica, Slovakia; lubomir.skladany@gmail.com; 10 School of Medicine, Zagreb University, Šalata ul. 2, 10000 Zagreb, Croatia; ivica.grgurevic@zg.htnet.hr (I.G.); tajana.filipec@gmail.com (T.F.-K.); 11 Division for Liver Diseases, Department of Gastroenterology, Dubrava University Hospital, Avenija Gojka Šuška 6, 10000 Zagreb, Croatia; 12 Department of Gastroenterology, Clinical Hospital Center Rijeka, Krešimirova ul. 42, 51000 Rijeka, Croatia; ivana.mikolasevic@gmail.com; 13 1st Department of General Surgery, Faculty of Medicine, Jagiellonian University Medical College, Jakubowskiego 2, 30-688 Kraków, Poland; tomasz.rogula@uj.edu.pl; 14 Health Education Campus, Case Western Reserve University School of Medicine, 9501 Euclid Ave, Cleveland, OH 44106, USA; 15 Department of Physiology, School of Medicine with the Division of Dentistry in Zabrze, Medical University of Silesia, Poniatowskiego 15, 40-055 Katowice, Poland

**Keywords:** coronavirus disease 2019 (COVID-19), fetuin-A, fibroblast growth factor 21 (FGF-21), irisin, pentraxin 3 (PTX3), severe acute respiratory syndrome coronavirus 2 (SARS-CoV-2)

## Abstract

Analysis of liver biopsy specimens showed that SARS-CoV-2 might have led to liver damage. This study aimed to evaluate the role of selected hepatokines and myokines in the development and progression of COVID-19. Seventy patients with laboratory-confirmed COVID-19 and 20 healthy volunteers were enrolled in the study. Irisin, pentraxin 3, fetuin-A, and FGF-21 serum concentrations and biochemical parameters were assessed using an immunoenzymatic method with commercially available enzyme immunoassay (EIA) or enzyme-linked immunosorbent assay (ELISA) kits. Serum fetuin-A concentrations were significantly decreased in COVID-19 patients compared to healthy volunteers. The serum concentration of FGF-21 was significantly increased in obese COVID-19 patients compared to overweight ones. Moreover, the FGF-21 level was higher in COVID-19 patients diagnosed with metabolic syndrome than in patients without metabolic syndrome. PTX3 concentration was higher in COVID-19 patients with higher HOMA-IR values than those with lower HOMA-IR values. COVID-19 patients with HOMA-IR ≤ 3 and >3 had significantly lower fetuin-A levels than the control group. Irisin concentration was significantly decreased in the HOMA-IR ≤ 3 COVID-19 subgroup when comparing with the control group. Lower levels of fetuin-A observed in COVID-19 patients despite higher HOMA-IR, CRP, and ferritin levels, pneumonia, patients requiring ICU care suggests that fetuin-A deficiency predisposes to more severe COVID-19 course. Upregulated pentraxin 3 may be used as a potential predictor of COVID-19 severity.

## 1. Introduction

Coronavirus (CoV) is a highly diverse family of single-stranded RNA viruses causing human and animal diseases [1]. Some human coronaviruses, such as HCoV-229E and HCoV-OC43 or HCoV-NL63 and HCoV-HKU1, cause seasonal respiratory infections usually associated with mild symptoms known as the ‘common cold’. On the contrary, other human coronaviruses such as severe acute respiratory syndrome coronavirus (SARS-CoV) and Middle East respiratory syndrome coronavirus (MERS-CoV) cause infections of bronchial epithelial cells, pneumocytes, and upper respiratory tract cells leading to severe, life-threatening respiratory pathologies and lung injuries. Lately, special attention is paid to SARS-CoV-2 causing new CoV-induced disease (COVID-19) responsible for the most recent ongoing pandemic [2].

The clinical spectrum of COVID-19 ranges from asymptomatic or subclinical forms to acute disease (occurred in 5% of cases) characterized by respiratory failure, multiorgan and systemic manifestations in terms of sepsis, septic shock, and multiple organ dysfunction syndromes (MODS). Most frequently, COVID-19 presents as an uncomplicated (mild) illness with symptoms of an upper respiratory tract viral infection, including mild fever, cough, sore throat, nasal congestion, malaise, headache, muscle pain. Additionally, the new loss of taste and smell, diarrhea, and vomiting usually are observed. The 14% of all cases include severe pneumonia and respiratory symptoms such as fever, severe dyspnea, respiratory distress, tachypnea (>30 breaths/min), and hypoxia (SpO_2_ < 90% on room air) [3]. The main complications reported in patients with SARS-CoV-2, in addition to the respiratory tract and lung pathologies, may include coagulopathy, cardiovascular complications, or liver enzyme abnormalities [4]. Severe disease, higher risk of hospitalization, and a poorer outcome are associated with risk factors such as advanced age and the presence of chronic conditions, especially cardiovascular and metabolic diseases like hypertension, diabetes, or obesity [5]. Recent evidence suggests that the common link between metabolic diseases and SARS-CoV-2 is the inflammatory response. Whereas SARS-CoV-2 infection induces activation of adaptive and innate immune responses, resulting in massive inflammation, the metabolic diseases cause low-grade inflammation with chronically elevated lower levels of inflammatory mediators that is impossible to resolve spontaneously. Low-grade inflammation induces (through the release of inflammatory cytokines) inhibition of insulin signaling, which, when associated with SARS-CoV-2 infection and the accompanying ‘cytokine storm’, starts a vicious cycle [4,5].

Recent studies showed that COVID-19 affects other organs. Some reports have indicated that more than half of COVID-19 patients show varying levels of liver disease and liver enzymes abnormalities [4]. Pathological study of liver biopsy specimens showed that SARS-CoV-2 might have led to liver damage by inducing microvesicular steatosis and mild lobular and portal activity [6]. Other studies found that the virus may bind to angiotensin-converting enzyme 2 (ACE2) on cholangiocytes, leading to their dysfunction and inducing a systemic inflammatory response causing liver injury. Consequently, local and systemic effects of inflammatory responses in SARS-CoV-2 infection can induce dysregulation of hetopokines and myokines production [6,7].

The above-described abnormalities raise our concern. We hypothesize that hepatokines and myokines play an essential role in the development and progression of COVID-19. We studied hepatokines (pentraxin 3 (PTX3), fibroblast growth factor 21 (FGF-21), fetuin-A), and myokine (irisin) serum concentrations in patients with SARS-CoV-2 infection. We analyzed the relationships between PTX3, FGF-21, fetuin-A, and irisin levels and COVID-19 severity, concomitant metabolic abnormalities, and liver injuries.

## 2. Materials and Methods

### 2.1. Study Population

Initially there were 100 patients recruited to the study; nevertheless, data of 70 patients were included into the analysis. Seventy patients (43 females and 27 males) with laboratory-confirmed COVID-19 and 20 healthy volunteers (10 females and 10 males) constituting the control group were enrolled in the study. The participants were tested for SARS-CoV-2 infection using a reverse-transcriptase–polymerase-chain-reaction (RT-PCR) assay done on material from a nasal or throat swab. Patients included in the study had mean diastolic blood pressure of 135.0 (121.5–150.0); mean systolic blood pressure of 85.0 (75.0–95.0). Hypertension was diagnosed in 23 COVID-19 patients, coronary artery disease in 10 patients. Seven patients were taking in proton pump inhibitors (PPIs); 6 patients, antiplatelet drugs (acetylsalicylic acid, clopidogrel); 3 patients, rivaroxaban; and 12 were treated with statin.

Information on present and past co-morbidities and current medication use was collected. The following exclusion criteria were applied: hepatitis B virus (HBV) and hepatitis C virus (HCV) infection, human immunodeficiency virus (HIV) co-infection, drug abuse, the neoplastic or thyroid disease, chronic renal failure, mental illnesses, chronic liver disease, and cirrhosis based on primary sclerosing cholangitis (PSC), primary biliary cholangitis (PBC), autoimmune hepatitis (AIH), and alcohol cirrhosis (AC).

The healthy volunteers (*n* = 20) reported no complaints about their health at the time of participation in the study, and no history of gastrointestinal or chronic liver diseases, smoking and alcohol intake, and systemic diseases.

For further analysis, the patients were divided according to their BMI (<30 and ≥30 kg/m^2^) or liver injury based on alanine aminotransferase (ALT) activity (<40 and ≥40 IU/L) and gamma-glutamyltransferase (GGT) activity (<50 and ≥50 IU/L). Insulin resistance was calculated according to the homeostasis model assessment for IR (HOMA-IR) based on the following formula: fasting insulin level (mUI/L) × fasting glucose level (mg/dL)/405. Because HOMA-IR was upregulated in most analyzed patients, the analysis was carried for patients divided into two subgroups: with HOMA-IR ≤3 and >3. The analysis was also run according to the presence of infection symptoms (fever, cough, or dyspnea) and gastrointestinal symptoms (diarrhea, nausea/vomiting, abdominal pain, or dysgeusia). Another analysis criterium was the presence of pneumonia. Assessment of COVID-19 severity was based on the necessity of ICU hospitalization.

### 2.2. Biochemical and Serological Assays

Serum samples were obtained from the peripheral blood collected at the hospital admission before any treatment was applied. Irisin, PTX3, fetuin-A, and FGF-21 serum concentrations were assessed in duplicate using an immunoenzymatic method with commercially available enzyme immunoassay (EIA) or enzyme-linked immunosorbent assay (ELISA) kits (BioVendor Laboratorni Medicina a.s., Brno, Czech Republic): Human Irisin ELISA Kit no RAGO18R (limit of detection: 1 ng/mL; intra-assay precision CV%: 4.863–8.193); inter-assay precision CV%: 8.027–9.719); PTX3 ELISA Kit no RD 191477200R (limit of detection: 22 pg/mL; intra-assay precision CV%: 2.9–3.9; inter-assay precision CV%: 6.0–8.4); Fetuin-A ELISA Kit no RD 191037100 (limit of detection: 0.104 ng/mL; intra-assay precision CV%: 2.2–3.6; inter-assay precision CV%: 3.1–6.3; FGF-21 ELISA Kit no 19108200R (limit of detection: 7 pg/mL; intra-assay precision CV%: 1.6–2.9; inter-assay precision CV%: 3.1–3.5). The remaining biochemical parameters (such as full blood count, renal function tests, serum ammonia, C-reactive protein (CRP) were measured using routine methods. The upper limit of normal ALT and AST activities was set at 40 IU/L and for gamma-glutamyltransferase (GGT) activity at 50 IU/L.

### 2.3. Ethical Statements

This study was carried out according to the guidelines of the Declaration of Helsinki of the World Medical Association and was approved by the Ethics Committee of the Jagiellonian University in Cracow (no. 1072.6120.157.2020). The participants provided written informed consent before the study started.

### 2.4. Statistical Analysis

The data were expressed as median with interquartile range (Me(IQR)). The Shapiro–Wilk test was used to evaluate the data distribution. The statistical significance of the difference in studied variables was tested using the Mann–Whitney *U*-test and ANOVA rank Kruskal–Wallis tests for independent groups. Correlations were analyzed with the Spearman rank correlation coefficient. Statistical significance was set at *p*-value < 0.05. The statistical analysis was performed with STATISTICA 10.0 (StatSoft Polska Sp z o.o., Cracow, Poland).

## 3. Results

### 3.1. Baseline Characteristics and Biochemical Parameters Analyzed in the Serum of COVID-19 Patients and Control Group

The baseline characteristics and laboratory data of 70 COVID-19 patients and 20 healthy volunteers are summarized in Table 1.

The median age of COVID-19 patients was 58.5 (49.0–67.0) years. The median BMI of the COVID-19 patients was 27.8 kg/m^2^ (25.6–31.4), which, according to the WHO obesity scale, is classified as overweight. Among the COVID-19 group, 13 of COVID-19 patients were obese, 57 patients with COVID-19 were non-obese and 19 were diagnosed with metabolic syndrome.

Gastrointestinal symptoms such as diarrhea, nausea/vomiting, abdominal pain, and dysgeusia were experienced by 15 patients. Forty-one patients had a cough, while 20 of them complained of dyspnea during the cough. Twenty-three COVID-19 patients had pneumonia, and 9 COVID-19 patients needed ICU hospitalization.

Serum fetuin-A concentrations were significantly decreased in COVID-19 patients compared to healthy volunteers (*p* < 0.001). There was no significant difference in serum irisin, FGF-21 and PTX3 level between COVID-19 patients and control group (*p* = 0.50; *p* = 0.90; *p* = 0.55; respectively, Table 1).

When comparing inflammatory markers, no significant difference in white blood cells (WBC) count was observed between these two groups (*p* = 0.06). However, when looking at acute-phase proteins (APPs), the significant increase of C-reactive protein (CRP) and ferritin in COVID-19 patients (*p* < 0.001 and *p* < 0.001, respectively) could be observed. Moreover, ALT and GGT activities were significantly higher in COVID-19 patients than in the control group (*p* = 0.02 and *p* = 0.003, respectively), while there were no significant differences in AST activity or bilirubin level (*p* = 0.38 and *p* = 0.25, respectively). Moreover, albumin levels were significantly decreased in COVID-19 patients (*p* = 0.02). 

### 3.2. Comparison of Biochemical Parameters Analyzed in the Blood of Male and Female COVID-19 Patients

Irisin, FGF-21, fetuin-A, and PTX3 serum levels did not differ significantly between male and female COVID-19 patients. We also did not observe any significant differences for inflammatory markers such as WBC, CRP, procalcitonin (PCT), or interleukin 6 (IL-6). On the other hand, ferritin level was significantly lower in male COVID-19 patients (*p* = 0.04). Surprisingly, female COVID-19 patients presented higher ALT levels (*p* = 0.01). No significant differences were found for AST, GGT, alkaline phosphatase (ALP) activities, albumin and iron levels, international normalized ratio (INR), and HOMA-IR values. Detailed results of all biochemical parameters analyzed in the blood of male and female COVID-19 patients are presented in Supplementary Table S1.

### 3.3. Comparison of Biochemical Parameters Analyzed in the Blood of COVID-19 Patients According to BMI and Metabolic Syndrome Presence

COVID-19 patients were divided into two subgroups: overweight (BMI < 30 kg/m^2^) and obese (≥30 kg/m^2^). Serum concentration of FGF-21 was significantly increased in obese COVID-19 patients compared to overweight ones (529.9 (379.3–708.3) vs. 157.6 (62.5–307.7) pg/mL; *p* = 0.001, respectively). There were no significant differences in fetuin-A, PTX3, and irisin levels in the two subgroups of patients (*p* = 0.48, *p* = 0.63, and *p* = 0.61, respectively). The levels of IL-6 and D-dimers were significantly higher in obese COVID-19 patients, although levels of other APPs did not differ between these two subgroups. Moreover, no significant differences between these two subgroups were noted for ALT, AST, GGT, ALP activities, bilirubin, albumin and iron levels, and INR and HOMA-IR values. Detailed results of biochemical parameters analyzed in the blood of overweight and obese COVID-19 patients are presented in Table 2.

COVID-19 patients with diagnosed metabolic syndrome comprised 27.1% of the study group. Similar to obese patients, there were no significant differences in the analyzed hepatokines and liver functions compared to COVID-19 patients without metabolic syndrome, except for FGF-21, which was higher in COVID-19 patients diagnosed with metabolic syndrome. Moreover, these two subgroups showed no differences in APPs (including IL-6), HOMA-IR, or serum iron levels (Table 3).

### 3.4. Comparison of Biochemical Parameters Analyzed in the Blood of COVID-19 Patients Presenting Different Infection Symptoms: Fever, Cough, and Dyspnea

Results of biochemical parameters analyzed in the blood of COVID-19 patients were analyzed depending on the presence of fever (body temperature > 38 °C). The analysis showed no significant differences in serum irisin, FGF-21, fetuin-A, or PTX3 levels between patients with and without fever. Acute-phase proteins levels did not differ between these two subgroups either. Moreover, no significant differences in tested liver function were found for patients with and without fever except for ALP activity. ALP activity was higher in COVID-19 patients showing no fever (56.0 (47.0–64.2) vs. 73.5 (57.0–91.0) IU/L; *p* = 0.001). No significant differences were found between these two subgroups of patients for the other analyzed parameters except for iron concentration, which was higher in COVID-19 patients that developed fever (19.1 (14.3–22.7) vs. 14.3 (8.80–19.7) μmol/L; *p* = 0.04).

Cough was observed in 41 out of 70 (59.5%) COVID-19 patients at the time of hospital admission, while concomitant dyspnea was presented in 20 (28.5%) of them. COVID-19 patients with a cough had significantly higher body temperature in comparison to the patients without the cough. 

Surprisingly, COVID-19 patients with intercurrent cough and dyspnea had, additionally, significantly lower ALT activity and significantly lower hemoglobin and fasting glucose levels than those with isolated cough. A detailed comparison of biochemical parameters analyzed in the blood of COVID-19 patients presenting cough and dyspnea or isolated cough only is shown in Supplementary Table S2.

### 3.5. Comparison of Biochemical Parameters Analyzed in the Blood of COVID-19 Patients with and without Gastrointestinal (GI) Symptoms 

When analyzing the study group according to the presence of GI symptoms, the inclusion criteria assumed the presence of at least two out of four following symptoms: diarrhea, nausea/vomiting, abdominal pain, dysgeusia. Fetuin-A, PTX3, FGF-21, and irisin did not differ significantly between COVID-19 patients with and without GI symptoms. However, symptomatic COVID-19 patients had significantly higher iron concentration and lower CRP level in comparison to those without GI problems (22.9 (21.3–23.4) vs. 16.1 (11.7–19.7) μmol/L; *p* < 0.001 and 1.2 (0.77–6.61) vs. 5.6 (2.2–21.3) mg/L; *p* = 0.03; respectively). There were no differences in other biochemical parameters analyzed in these two subgroups of COVID-19 patients.

### 3.6. Comparison of Biochemical Parameters Analyzed in the Blood of COVID-19 Patients with and without Liver Injury Based on ALT and GGT Activities

The liver injury in COVID-19 patients was assessed based on ALT activity, and the study group was divided accordingly (<40 and ≥40 IU/L). There were no significant differences in irisin, FGF-21, fetuin-A, and PTX3 levels and other biochemical parameters between the groups of COVID-19 patients with different ALT activity. COVID-19 patients with higher ALT activity also presented a statistically significant increase in other hepatic parameters and HOMA-IR value. No significant differences in APPs, iron, and lipid fractions (total and LDL-cholesterol or triglycerides) were observed between these subgroups. The detailed results of biochemical parameters analyzed in the blood of COVID-19 patients in the distinguished subgroups are presented in Supplementary Table S3.

When dividing COVID-19 patients according to GGT activity level (<50 IU/L and ≥50 IU/L), the patients with elevated GGT activity presented significantly decreased concentrations of fetuin-A (221.9 (182.1–256.3) vs. 258.8 (219.9–310.9) µg/mL; *p* = 0.004, respectively). There were no significant differences in irisin, FGF-21, and PTX3 levels in the groups of COVID-19 patients with different GGT activity. The COVID-19 patients with higher GGT activity presented statistically significantly higher levels of their hepatic parameters, except for bilirubin concentration. COVID-19 patients with elevated GGT activity also had significantly elevated HOMA-IR values. Contrary to COVID-19 patients with increased ALT activity, COVID-19 patients with higher GGT activity had significantly higher concentrations of APPs, such as CRP or ferritin, and higher D-dimers levels. On the other hand, this subgroup of COVID-19 patients presented significantly lower albumin concentration Table 4.

### 3.7. Comparison of Biochemical Parameters Analyzed in the Blood of COVID-19 Patients with and without Pneumonia 

All COVID-19 patients were assessed for clinical or radiological signs of pneumonia at admission to the hospital. Pulmonary inflammation was diagnosed in 23 out of 70 (32.8%) COVID-19 patients. Serum fetuin-A concentrations were significantly decreased in COVID-19 patients with pneumonia (217.4 (188.3–248.8) vs. 256.3 (218.9–285.7) µg/mL; *p* < 0.001, respectively). We did not observe any significant differences in serum irisin, FGF-21, and PTX3 concentrations. COVID-19 patients with pneumonia had significantly higher GGT activity (*p* = 0.003). However, the activities of other liver enzymes and bilirubin levels remained unchanged. As expected, the markers of inflammation, such as CRP, IL-6, or ferritin, were significantly higher in COVID-19 patients with pneumonia. These patients also presented lower serum total protein and albumin concentrations. HOMA-IR values did not differ significantly between these two groups of COVID-19 patients. The detailer results for the two distinguished groups of COVID-19 patients are presented in Table 5.

### 3.8. Comparison of Biochemical Parameters Analyzed in the Blood of COVID-19 Patients Requiring and Nonrequiring Intensive Care Unit (ICU) Hospitalization

Among COVID-19 patients, 15% presented severe clinical condition, which required treatment in ICU. COVID-19 patients admitted to ICU presented higher PTX3 and lower fetuin-A concentration than patients not requiring hospitalization. COVID-19 patients admitted to ICU had significantly elevated GGT activity and inflammatory markers levels (WBC count, CRP, IL-6, ferritin) than the rest of COVID-19 patients. Additionally, they had significantly higher triglycerides, D-dimer levels, lactate dehydrogenase (LDH) activity, PLT count, HOMA-IR value, and decreased HDL-cholesterol and albumin concentrations. A detailed comparison of biochemical parameters analyzed in the blood of COVID-19 patients requiring and nonrequiring ICU care is shown in Table 6.

### 3.9. Comparison of Biochemical Parameters Analyzed in the Blood of COVID-19 Patients with Different Iron and Ferritin Levels

We also analyzed the results according to COVID-19 patients’ serum iron (≤16.8 and >16.8 μmol/L) and ferritin (≤250.0 and >250.0 µg/L) concentrations. The results form 64 patients were included in this analysis. The details of these analyses are presented in Table 7 and Table 8.

COVID-19 patients with non-elevated ferritin levels had significantly higher fetuin-A concentrations. COVID-19 patients with higher ferritin levels showed increased FGF-21 and PTX3 concentrations. They also showed significantly higher APPs, hepatic parameters (except for ALP activity), HOMA-IR values, and LDH activity with simultaneous decrease in albumin, HDL- and LDL-cholesterol concentrations.

Patients with iron deficiency had significantly lower irisin and significantly lower PTX3 concentration. We did not observe any differences in FGF-21 concentration between COVID-19 patients with and without iron deficiency. The iron deficiency subgroup also had a significantly higher concentration of APPs (CRP and IL-6), ALP activity, and total and LDL-cholesterol levels. 

### 3.10. Comparison of Biochemical Parameters Analyzed in the Blood of COVID-19 Patients with Different HOMA-IR Values

Analyzing serum concentrations of irisin, fetuin-A, and FGF-21 were the same in COVID-19 patients with various insulin sensitivity, HOMA-IR value ≤3 and >3, (*p* = 0.80, *p* = 0.19 and *p* = 0.40, respectively), but PTX3 concentration was higher in COVID-19 patients with higher HOMA-IR value than these with lower HOMA-IR value (3038.6 (2310.2–4875.4) vs. 2097.7 (1825.2–3099.4) pg/mL; *p* = 0.02). COVID-19 patients with HOMA-IR ≤ 3 had significantly lower AST, GGT, and ALP activities than those with HOMA-IR > 3 (22.0 (17.0–26.7) vs. 33.5 (20.5–51.0) IU/L, *p* = 0.02, 27.0 (14.0–35.0) vs. 71.0 (34.7–266.7) IU/L, *p* < 0.001 and 56.0 (52.2–64.2) vs. 67.5 (61.0–91.0) IU/L, *p* = 0.01; respectively). Other parameters describing liver function (ALT, LDH, total bilirubin, INR, albumin) did not differ between these two subgroups. We also observed significantly lower levels of ferritin and IL-6 between COVID-19 patients with HOMA-IR ≤ 3 (167.0 (105.0–526.0) vs. 474.0 (231.2–834.5) µg/L, *p* = 0.04 and 1.50 (1.50–3.26) vs. 5.19 (1.50–10.45) pg/mL, *p* = 0.01; respectively), although levels of other APPs (CRP, PCT) did not differ between these two subgroups. Moreover, no significant differences between COVID-19 patients with various insulin sensitivity were found for all analyzed lipid parameters.

Additional comparison of COVID-19 patients with different insulin sensitivity and the control group demonstrated that COVID-19 patients with HOMA-IR ≤3 and >3 had significantly lower fetuin-A levels than the control group (256.7 (230.6–314.0) vs. 333.5 (303.0–371.4) µg/mL; *p* = 0.001 and 231.2 (190.7–276.1) vs. 333.5 (303.0–371.4) µg/mL; *p* < 0.001, respectively). Irisin concentration was significantly decreased in the HOMA-IR ≤ 3 COVID-19 subgroup when comparing with the control group (5.2 (4.6–5.7) vs. 6.5 (5.5–7.8) μmol/L; *p* = 0.008), while no difference was found between COVID-19 patients with HOMA-IR > 3 and control group (4.9 (3.7–6.4) vs. 6.5 (5.5–7.8) μmol/L; *p* = 0.07). 

### 3.11. Comparison of Biochemical Parameters Analyzed in the Blood of COVID-19 Patients Depending on Their Lipid Disorders 

We observed that irisin concentration was significantly increased in COVID-19 patients with increased total cholesterol (5.94 (4.37–8.04) vs. 5.07 (3.63–5.77) μmol/L; *p* = 0.03). There were no significant differences in irisin levels between COVID-19 patients with increased and normal LDL-cholesterol, HDL-cholesterol and triglycerides levels (*p* = 0.09, *p* = 0.09 and *p* = 0.19). No such differences were found for FGF-21, fetuin-A, PTX3 concentrations. The group of COVID-19 patients with higher total and LDL-cholesterol levels was characterized by significantly lower CRP and IL-6 serum concentrations and significantly elevated PLT count and iron level. 

### 3.12. Correlations between Hepatokines Concentrations and Biochemical Parameters Analyzed in the Blood of COVID-19 Patients

We observed significant positive correlations between irisin concentration and total, LDL, and HDL cholesterol concentrations (r = 0.39, *p* = 0.001, r = 0.30, *p* = 0.01 and r = 0.33, *p* = 0.01). We also found significant positive correlations between irisin concentration and PLT count, iron (Table 9), and protein (r = 0.41, *p* < 0.001) concentrations. On the other hand, it negatively correlated with CRP (Table 9), creatinine, and PT (prothrombin time) (r = −0.24, *p* = 0.04 and r = −0.41, *p* = 0.04, respectively). FGF-21 serum concentration positively correlated with BMI (r = 0.58, *p* < 0.001), LDH (r = 0.35, *p* = 0.006), fasting glucose (r = 0.32, *p* = 0.01), ferritin and IL-6 concentrations. PTX3 concentrations correlated postively with LDH activity (r = 0.39, *p* = 0.001) and APPs (CRP, IL-6, and ferritin) concentrations (Table 9) and negatively with PLT count, iron, albumin (Table 9), and protein (r = −0.35, *p* = 0.005) concentrations. Fetuin-A serum concentrations correlated negatively with LDH (r = −0.43, *p* < 0.001), GGT activities, CRP, IL-6, and ferritin concentration and positively with iron, albumin (Table 9), protein (r = 0.52, *p* < 0.001), total (r = 0.34, *p* = 0.006) and LDL-cholesterol (r = 0.31, *p* = 0.01) concentrations.

We also observed significant correlations between the analyzed hepatokines. PTX3 correlated positively with FGF-21 and negatively with irisin and fetuin-A. At the same time, fetuin-A was positively associated with irisin and negatively with FGF-21 (Table 9).

## 4. Discussion

Subjects suffering from COVID-19 express a range of similar symptoms such as lymphopenia, hypercoagulability, and cytokine storm (elevation in IL-6, CRP, TNF, MCP1, IL-1β levels, and others) [8]. The cytokines storm can influence the pathophysiological processes leading to septic shock and multiple organ failure. Elevated levels of IL-6 are a good marker of poor outcomes in patients with severe COVID-19 with pneumonia and acute respiratory distress syndrome (ARDS) [5]. That is why it is essential to identify and combat hyperinflammation with accepted therapies to reduce organ damage and mortality [5]. To date, several potential anti-inflammatory therapies are under scrutiny, including glucocorticoids, IL-6 antagonists, and JAK inhibitors [5].

Obesity and insulin resistance are core components of metabolic syndrome and significant risk factors for severe disease development, higher risk of hospitalization, and a poorer outcome in patients with SARS-CoV-2 infection. Some studies attempted to reveal the detailed mechanism linking metabolic abnormalities to their complications. It has been reported that in patients with diagnosed metabolic syndrome, the abnormalities in the circulating levels of irisin and its gene expression are related to BMI and markers of insulin sensitivity [9]. As mentioned above, overweightness and obesity are scientifically proven risk factors of more severe course of COVID-19 and, in consequence, of its higher mortality [10,11]. Moreover, patients with T2DM are more prone to develop COVID-19 disease and complications such as ARDS and death [12]. This information seems crucial as the same chronic conditions are directly correlated with fluctuations in the analyzed hepatokines.

So far, only a few studies have analyzed the influence of hepatokines on the development or the course of SARS-CoV-2 infection. Our study is the first to show significantly lower fetuin-A levels in the serum of COVID-19 patients. It may indicate the crucial role of this hepatokine in COVID-19 pathogenesis. In the literature, we can find mentions of the pleiotropic role of fetuin-A in diverse biological processes, including immune response regulation and inflammation [13,14]. Most of them characterized fetuin-A as a liver-derived negative APP in systemic inflammatory diseases because its serum concentration decreases during the acute inflammatory response and normalizes when the infection is successfully treated. Previous publications presented that among patients with other inflammatory diseases, such as pancreatitis [15], chronic kidney diseases [16], and rheumatoid arthritis [17], serum fetuin-A levels decreased by 20–30%. Moreover, most of these studies suggested negative correlations between circulating fetuin-A level and cytokines and inflammatory markers, such as IL-6, CRP, procalcitonin, or WBC [18,19], which is consistent with results presented in our study. Wang and Sama [15] tried to elucidate the direct mechanism of fetuin-A downregulation. According to them, innate immune cells (such as macrophages) sequentially release early (e.g., tumor necrotic factor, TNF, and interferon γ, IFN-γ) and late (e.g., high mobility group box 1, HMGB1) proinflammatory mediators. The hepatic expression of fetuin-A is negatively regulated by several early proinflammatory cytokines, such as TNF, IL-1, IL-6, and IFN-γ, consequently allowing propagation of a rigorous inflammatory response manifested by excess accumulation of late proinflammatory mediators, such as HMGB1. In contrast, HMGB1 elevates hepatic fetuin-A expression levels by two to three times, thereby restoring circulating fetuin-A levels during a late stage of the inflammation process. We also hypothesize that lower fetuin-A levels could influence disease course and be associated with the severity of COVID-19 because we observed a significant decrease in its concentration among patients with confirmed pneumonia and these requiring ICU care. What is interesting, according to another study, is that downregulation of fetuin-A is associated with severity and exacerbation frequency of other respiratory diseases—chronic obstructive pulmonary disease (COPD) [20]. Minas et al. assessed the levels of fetuin-A in one hundred COPD patients in stable condition and on exacerbation. COPD patients presented lower levels of fetuin-A compared to the control group (*p* < 0.001). COPD patients with GOLD stage IV had lower fetuin-A levels than with stages I, II and III (*p* < 0.05). Moreover, according to them, fetuin-A may represent a potential biomarker for the prediction and evaluation of exacerbations in patients with COPD because it is markedly reduced at the onset of an ECOPD compared to baseline, and patients with low fetuin-A values had a two-fold risk for a COPD exacerbation during the one-year follow-up period. Similarly, decreased fetuin-A is associated with increased disease activity in Crohn’s disease and ulcerative colitis [21]. Fetuin-A is believed to play a protective role in inflammatory bowel disease (IBD) by acting as an endogenous inhibitor of meprin-α, which plays an essential role in IBD pathogenesis by activating inflammatory cytokines [22] and protecting against intestinal inflammation by inhibiting HMGB1 release [23]. The above information implies the possible role of fetuin-A decrease in the development of cytokine storm in SARS-CoV-2 infection. Subsequently, it suggests that fetuin-A deficiency predisposes to a more severe COVID-19 course. Moreover, our results show that this reduction is independent of such factors as sex, metabolic disorders, lipids level, BMI, respiratory symptoms, or liver injury, implying a direct influence of COVID-19 infection on hepatokine secretion.

As mentioned above, our study, for the first time, showed significantly lower levels of serum fetuin-A in COVID-19 patients. We did not find any significant differences for the rest of the analyzed hepatokines concentrations, even though other studies presented data proving that these molecules can modulate the course of SARS-CoV-2 infection. There is only one study analyzing the influence of irisin on COVID-19 [24]. According to de Oliveira et al., irisin presented a very positive regulatory effect on diverse genes related to the COVID-19 outcome in the adipose tissue. They reported that irisin reduced the expression of genes implicated in elevated viral infection and increased the expression of genes involved in blocking the virus–cell cleavage [24]. Unfortunately, it is not easy to compare this data to our results because the study did not compare irisin concentrations. 

Ajaz et al. performed a study analyzing the levels of FGF-21 among healthy control (HC) (*n* = 9), patients with COVID-19 (*n* = 7), and patients with a chest infection (*n* = 7) [25]. Contrary to our observations, they demonstrated an increase in FGF-21 levels in patients with COVID-19 compared to HC (*p* < 0.001). They noticed a trend of high levels of FGF-21 and an increase in COVID-19 severity among patients who were admitted to ICU (*p* < 0.001) and died due to COVID-19 (*p* = 0.001). Although, a relatively small number of patients in Ajaz’s study is an essential factor that may affect the final results and explain the difference compared to our study. Regarding a hypothetical relationship of FGF-21 with the severity of COVID-19 course in our study, we could discern significantly positive correlation in this hepatokine levels and such inflammation markers as ferritin or IL-6. 

Up to this date, several studies analyzed PTX3 among patients with confirmed SARS-CoV-2 infection. PTX3 plays a vital role in innate immunity and inflammation. It participates in innate resistance to fungal, bacterial, and viral infections. PTX3 acts as an opsonin for pathogens and regulates the inflammatory response by modulating complement activity, recruiting inflammatory cells through interacting with the adhesion molecule P-selectin [26]. Brunetta et al. [27] reported increased PTX3 plasma concentrations in 96 patients with COVID-19. Similar to our study, they found a significant correlation of PTX3 with APPs: CRP, PCT, IL-6, or ferritin. Moreover, in multivariable analysis, PTX3 emerged as a strong independent predictor, better than conventional markers of inflammation, of 28-day mortality in hospitalized COVID-19 patients [27]. The prognostic significance of PTX3 abundance for mortality was confirmed in a second independent cohort (54 patients). Moreover, other studies emphasize the prognostic role of PTX3 levels and its relation to COVID-19 severity. Tong et al. [28] indicated a positive relationship between PTX3 and intensification of lung lesions expressed as chest CT imaging score (r = 0.418, *p* = 0.008), and length of hospital stay (r = 0.486, *p* = 0.002). Genç et al. [29] presented significantly increased levels of this hepatokine in nonsurvivors than survivors with confirmed COVID-19, suggesting that PTX3 is an indicator of inflammation and death due to COVID-19 pneumonia. The above results seem consistent with our study, which showed that in COVID-19 patients requiring ICU care, serum PTX3 concentration was significantly increased than patients not requiring it. We also hypothesize that higher PTX3 levels could influence disease course and be associated with the necessity of hospitalization due to SARS-CoV-2 virus infection, even though we did not observe any differences in this hepatokine concentration between patients with and without pneumonia. Increased concentrations of PTX3 in COVID-19 patients may reflect failed negative regulation of uncontrolled inflammation—cytokine storm [27].

The onset of COVID-19 usually manifests by respiratory symptoms such as cough or dyspnea and fever. Although other publications have indicated that more than half of COVID-19 patients showed varying levels of liver disease [30], our study showed that COVID-19 patients had significantly elevated ALT and GGT activities compared to the control group. On the other hand, we found no significant differences in AST activity or bilirubin level. Moreover, albumin levels were significantly decreased in infected patients. Our results seem to be consistent with other reports indicating abnormal liver function observed in cases of COVID-19, manifesting mainly as isolated elevated serum transaminases, GGT, and LDH activities [31]. Meta-analysis performed by Rodriguez-Morales et al. [32], investigating liver function test (LFT) abnormalities, pointed to an accumulated elevation of AST in 33.3% and ALT in 24.1% of cases. Our study has also been investigating the association between LFT abnormalities and the severity of infection. GGT activity was significantly increased among patients with pneumonia. However other liver enzymes’ activities and bilirubin levels remained unchanged. Moreover, according to our data, COVID-19 patients requiring ICU care had significantly elevated GGT and LDH activity. Similarly, a study by Cai et al. [33] showed that patients with abnormal LFT, especially in hepatocyte type or mixed type, presented a significantly higher risk of developing severe pneumonia. Moreover, studies on Wuhan patients showed that liver injury rates were higher in ICU patients and nonsurvivors, suggesting that liver injury is most likely to occur in critically ill patients [34,35,36]. On the contrary, according to Vespa et al. [37], markers of hepatocellular injury (AST or ALT), as well as GGT and total bilirubin, cannot be used as predictors of death or admittance to the ICU. The only predictive factor associated with health deterioration was ALP activity higher than 150 IU/L.

In our study, patients with fever presented significantly lower ALP activity than patients with normal body temperature. Moreover, COVID-19 patients with intercurrent cough and dyspnea had significantly lower ALT activity than patients with isolated cough. Contrarily, Cai et al. [33] and Wang et al. [4] presented a significantly higher frequency of cough as COVID-19 initial symptom in patients with elevated liver enzymes activities. As mentioned above, we divided COVID-19 patients according to liver injury based on elevated ALT and GGT activity. In the group of patients with higher GGT levels, we observed a statistically significant decrease in fetuin-A concentration. No other significant differences in the analyzed hepatokines were found in patients with liver injury. So far, studies investigating the circulating fetuin-A levels in patients with liver diseases of various etiology presented inconsistent results. According to two recent meta-analyses [38,39], the circulating levels of fetuin-A in patients with non-alcoholic fatty liver disease (NAFLD) were significantly higher than in healthy controls. Moreover, one of the meta-analyses showed that a higher level of fetuin-A was more pronounced in the non-alcoholic steatohepatitis (NASH) group than in the NAFLD group [39]. Contrarily, Cui et al. [40], which performed a study on patients with NAFLD in the Chinese population, showed that serum fetuin-A concentration was significantly lower than that in the control group (0.27 ± 0.17 vs. 0.32 ± 0.12 g/L, *p* < 0.05). In their study, 158 subjects were divided into four groups (controls, mild, moderate, and severe NAFLD) according to serum ALT level. Compared to controls (0.32 ± 0.12 g/L), mild NAFLD (0.24 ± 0.16 g/L, *p* < 0.05) and moderate NAFLD (0.25 ± 0.17 g/L, *p* < 0.05) patients had significantly lower levels of fetuin-A. The study showed that serum fetuin-A levels tended to increase with the severity of NAFLD. 

Considering the above-presented data seems evident that LFTs alterations are common in hospitalized COVID-19 patients. The etiology of liver injury is probably multifactorial and associated with drug-induced liver injury, and the secondary liver injury might be induced by systemic inflammatory response syndrome or hypoxia. It is still unclear if laboratory LFTs abnormalities have any prognostic value for predicting the development of the COVID-19 course.

To date, only a few studies have investigated the influence of insulin resistance on the course of COVID-19 infection. The adverse impact of higher insulin resistance on the severity and mortality of COVID-19 patients has been proved by most of them, suggesting that chronic inflammation is a key risk factor in this group of patients [41,42]. Finucane and Davenport [43] argue that the state of insulin resistance and elevated insulin levels that are driving increased ACE2 expression in lung epithelial cells are responsible, at least in part, for aggravating disease severity. On the other hand, COVID-19 can deteriorate insulin resistance in people with T2DM and T1DM via inducing a proinflammatory milieu that can further lead to lowering insulin sensitivity. In addition, Pal and Bhadada hypothesized that one of the mechanisms leading to impaired insulin sensitivity in SARS-CoV-2 infection is increased serum levels of fetuin-A [44]. However, our results indicated significant downregulation of fetuin-A in COVID-19 patients. Furthermore, we found no difference in fetuin-A levels in patients with different HOMA-IR values. As mentioned above, COVID-19 patients with HOMA-IR > 3 had significantly elevated levels of IL-6 and ferritin, which could be linked to a chronic inflammatory state in this group. We also observed significantly higher PTX3 concentration in COVID-19 patients with HOMA-IR > 3. Previous studies presented inconsistent data on PTX3 connection to insulin resistance. Jylhävä et al. [45], who investigated the PTX3 relationship with cardiovascular risk factors, did not indicate any statistically significant correlation between PTX3 and HOMA-IR values. Contrary to our research, Chu et al. [46] presented negative association between circulating PTX3 levels with fasting insulin (r = −0.336, *p* = 0.012) and homeostasis model assessment of insulin resistance (HOMA-IR) (r = −0.334, *p* = 0.014) among 57 overweight or obese children. The potential mechanism linking PTX3 levels and insulin resistance could be the phosphatidylinositol 3-kinase (PI3K) and downstream signaling pathways, including protein kinase B. Recent studies have found PI3K/Akt activation-dependent expression of PTX3 in endothelial cells [47], suggesting a possible link between PTX3 and insulin signaling.

Our research has several limitations, the first of them being the study group consisting of a relatively small number of patients. The second limitation of the study is that it assesses liver injury or cholestasis based on liver markers such as ALT, AST, or GGT. However, the exact stage of liver injury was not evaluated. Furthermore, the study included a relatively small number of obese patients. Another limitation of the presented study is the lack of hepatokines and other laboratory parameters evaluation during subsequent days of hospitalization. It prevents from analyzing hepatokines fluctuations and from assessing their relationship with severity of infection. The last limitation study was the lack of liver imaging.

## 5. Conclusions

Pointing to proinflammatory and insulin impairing the action of fetuin-A, its levels were significantly lower in COVID-19 patients despite higher HOMA-IR, CRP, and ferritin levels. Even more surprising was the significant reduction of fetuin-A level in COVID-19 patients with pneumonia, patients requiring ICU care, or those with higher ferritin levels and HOMA-IR values. It suggests that fetuin-A deficiency predisposes to a more severe COVID-19 course, and glucose metabolism abnormalities and their measurements may be the additional marker of COVID-19 severity. Upregulated PTX3 may also predict COVID-19 severity because COVID-19 patients with significantly elevated PTX3 concentrations more frequently required ICU care. Impaired liver function, assessed with GGT activity, was associated with serum fetuin-A depletion. GGT activity occurred as a predictive factor of a more severe course of COVID-19, including pneumonia development and ICU hospitalization. Pointing to all the results, the exact role of the analyzed hepatokines and liver injury in SARS-CoV-2 infection requires additional studies.

## Figures and Tables

**Table 1:** The baseline characteristics and laboratory data of patients and healthy volunteers.

Parameters	COVID-19 Patients (*n* = 70)	Healthy Volunteers (*n* = 20)	*p*
Age [years]	58.5 (49.0–67.0)	50.0 (43.0–58.5)	0.02
BMI [kg/m^2^]	27.8 (25.6–31.4)	29.7 (25.3–32.4)	0.67
FGF-21 [pg/mL]	261.3 (96.3–507.0)	276.3 (228.9–408.6)	0.90
PTX3 [pg/mL]	2337.7 (1935.1–3211.5)	2030.9 (1767.4–3499.5)	0.55
Fetuin-A [µg/mL]	243.4 (195.0–275.8)	333.4 (302.9–371.3)	<0.001
Irisin [ng/mL]	5.39 (3.85–6.69)	6.54 (5.53–7.83)	0.06
WBC [10^3^/µL]	5.88 (4.51–6.43)	6.78 (5.14–7.49)	0.05
RBC [10^6^/µL]	4.30 (4.07–4.51)	4.66 (4.51–4.99)	0.001
HCT [%]	38.2 (36.4–40.5)	41.5 (39.6–43.4)	0.002
HGB [mg/dL]	12.7 (12.3–13.3)	14.5 (13.2–14.6)	0.001
PLT [10^3^/µL]	246.5 (202.0–333.0)	223.5 (197.0–259.0)	0.17
CRP [mg/L]	5.53 (2.08–18.2)	0.80 (0.50–1.58)	<0.001
Ferritin [µg/L]	210.0 (114.7–487.0)	16.5 (13.0–19.6)	<0.001
ALT [IU/L]	34.0 (20.0–52.5)	21.0 (16.0–25.7)	0.02
AST [IU/L]	25.0 (17.5–43.0)	24.0 (20.0–26.5)	0.38
GGT [IU/L]	33.5 (20.0–62.5)	14.0 (10.7–24.2)	0.003
Bilirubin [µmol/L]	6.16 (4.57–8.17)	7.35 (5.40–9.70)	0.25
Fasting glucose [mmol/L]	5.27 (4.70–6.01)	5.50 (4.05–5.73)	0.59
Urea [mmol/L]	5.39 (4.20–6.21)	5.67 (5.45–6.94)	0.16
Creatinine [µmol/L]	66.3 (58.9–87.2)	85.2 (73.3–95.4)	0.01
Cholesterol [mmol/L]	4.80 (3.70–5.57)	5.26 (3.54–6.54)	0.49
Triglycerides [mmol/L]	1.53 (1.26–1.86)	1.83 (1.04–2.03)	0.97
HDL [mmol/L]	1.01 (0.84–1.15)	1.12 (1.06–1.32)	0.12
LDL [mmol/L]	2.48 (1.68–3.32)	2.65 (1.48–4.37)	0.76
INR	0.96 (0.92–1.00)	1.06 (1.00–1.12)	0.009
Total protein [g/L]	65.0 (62.0–71.0)	65.5 (63.0–71.0)	0.75
Albumin [g/L]	40.0 (34.2–43.0)	43.0 (40.0–45.0)	0.02
HOMA-IR	2.42 (1.85–2.85)	1.66 (0.61–2.67)	0.13
Saturation %	97 (95–98)	97 (96–97)	0.92

Abbreviations: ALT—alanine transaminase, AST—aspartate transaminase, BMI—body mass index, CRP—C-reactive protein, GGT—gamma-glutamyl-transferase, HCT—hematocrit, HDL—high-density lipoprotein, HGB—hemoglobin, INR—international normalized ratio, LDL—low-density lipoprotein, WBC—white blood cells, PLT—platelet count, RBC—red blood cells.

**Table 2:** Comparison of biochemical parameters analyzed in the blood of COVID-19 patients with and without obesity.

Parameters	Non-Obese Patients (*n* = 57)	Obese Patients (*n* = 13)	*p*
FGF-21 [pg/mL]	157.6 (62.5–307.7)	529.9 (379.3–708.3)	0.001
PTX3 [pg/mL]	2317.8 (1484.0–2983.6)	2258.4 (2028.7–3126.4)	0.63
Fetuin-A [µg/mL]	240.0 (202.9–270.6)	265.6 (205.6–321.9)	0.48
Irisin [ng/mL]	5.66 (4.57–6.74)	5.31 (3.35–6.69)	0.61
WBC [10^3^/µL]	5.93 (4.68–6.62)	6.24 (5.50–7.65)	0.21
HGB [mg/dL]	12.9 (12.4–13.2)	12.3 (11.3–13.8)	0.21
PLT [10^3^/µL]	283.0 (205.0–338.0)	253.0 (209.0–303.2)	0.62
CRP [mg/L]	5.96 (1.54–7.38)	4.39 (3.10–5.88)	1.00
IL-6 [pg/mL]	1.50 (1.50–3.72)	5.38 (2.79–9.03)	0.009
Ferritin [µg/L]	175.0 (121.2–258.2)	230.5 (92.6–779.5)	0.25
Iron [µmol/L]	20.1 (12.8–23.6)	16.1 (13.2–20.4)	0.26
ALT [IU/L]	31.0 (15.0–56.0)	21.0 (19.7–43.0)	0.78
AST [IU/L]	22.5 (15.0–45.5)	23.0 (17.2–24.5)	0.92
GGT [IU/L]	33.0 (15.5–65.5)	26.0 (20.2–246.5)	0.50
ALP [IU/L]	64.0 (50.7–75.7)	57.0 (56.0–69.0)	1.00
Bilirubin [µmol/L]	6.09 (3.94–6.92)	6.80 (5.02–9.48)	0.17
INR	0.95 (0.93–0.95)	0.97 (0.92–0.98)	0.68
Albumin [g/L]	41.0 (37.0–43.0)	40.0 (33.5–43.2)	0.43
HOMA-IR	2.24 (1.80–2.65)	2.01 (1.17–3.54)	0.93
Saturation %	98 (95–98)	96 (87.5–97.25)	0.03

Abbreviations: ALT—alanine transaminase, AST—aspartate transaminase, BMI—body mass index, CRP—C-reactive protein, GGT—gamma-glutamyl-transferase, HCT—hematocrit, HDL—high-density lipoprotein, HGB—hemoglobin, INR—international normalized ratio, LDL—low-density lipoprotein, WBC—white blood cells, PLT—platelet count, RBC—red blood cells.

**Table 3:** Comparison of biochemical parameters analyzed in the blood of COVID-19 patients with and without metabolic syndrome.

Parameters	Patients with Metabolic Syndrome (*n* = 19)	Patients without Metabolic Syndrome (*n* = 51)	*p*
FGF-21 [pg/mL]	408.6 (166.5–585.6)	187.0 (87.1–416.4)	0.03
PTX3 [pg/mL]	2375.8 (2112.25–3611.4)	2313.4 (1868.6–3199.0)	0.52
Fetuin-A [µg/mL]	238.8 (194.3–265.5)	249.1 (201.8–276.0)	0.64
Irisin [ng/mL]	5.33 (3.92–7.36)	5.39 (3.57–6.34)	0.55
WBC [10^3^/µL]	6.16 (5.48–7.43)	5.65 (4.33–6.24)	0.04
HGB [mg/dL]	12.9 (12.4–13.0)	12.7 (12.2–13.7)	0.94
PLT [10^3^/µL]	314.5 (227.0–403.0)	231.5 (188.0–292.0)	0.003
CRP [mg/L]	4.39 (2.13–5.78)	5.88 (1.99–29.8)	0.31
IL-6 [pg/mL]	3.42 (1.50–3.98)	2.89 (1.50–7.56)	0.79
Ferritin [µg/L]	307.0 (98.2–634.5)	183.5 (123.0–311.0)	0.17
Iron [µmol/L]	16.4 (11.3–22.7)	17.4 (11.1–21.7)	0.93
ALT [IU/L]	43.0 (26.5–81.0)	32.5 (18.5–52.5)	0.11
AST [IU/L]	31.0 (20.2–50.0)	25.0 (17.0–40.2)	0.33
GGT [IU/L]	34.0 (18.7–65.0)	32.5 (20.5–63.0)	0.68
ALP [IU/L]	61.0 (55.2–79.5)	64.5 (54.0–85.0)	0.65
Bilirubin [µmol/L]	5.97 (4.32–7.03)	6.40 (4.70–8.62)	0.21
INR	0.97 (0.92–1.04)	0.96 (0.92–1.00)	0.80
Albumin [g/L]	40.0 (34.0–42.0)	40.0 (34.7–43.2)	0.44
HOMA-IR	3.59 (2.24–9.41)	2.16 (1.80–2.65)	0.007
Saturation %	96.5 (93.5–98)	97 (96–98)	0.54

Abbreviations: ALT—alanine transaminase, AST—aspartate transaminase, BMI—body mass index, CRP—C-reactive protein, GGT—gamma-glutamyl-transferase, HCT—hematocrit, HDL—high-density lipoprotein, HGB—hemoglobin, INR—international normalized ratio, LDL—low-density lipoprotein, WBC—white blood cells, PLT—platelet count, RBC—red blood cells.

**Table 4:** Comparison of biochemical parameters analyzed in the blood of COVID-19 patients according to GGT activity.

Parameters	GGT Activity <50 IU/L (*n* = 36)	GGT Activity ≥50 IU/L (*n* = 34)	*p*
FGF-21 [pg/mL]	163.9 (80.3–538.5)	275.5 (95.5–504.1)	0.65
PTX3 [pg/mL]	2272.7 (1876.3–2685.7)	2875.4 (2126.9–4034.5)	0.05
Fetuin-A [µg/mL]	258.8 (219.9–310.9)	221.9 (182.1–256.3)	0.004
Irisin [ng/mL]	5.35 (4.20–5.79)	5.31 (3.53–7.71)	0.98
WBC [10^3^/µL]	5.98 (5.10–6.61)	4.97 (4.16–6.44)	0.21
HGB [mg/dL]	12.8 (12.3–13.2)	12.7 (11.9–13.5)	0.69
PLT [10^3^/µL]	232.0 (202.2–307.7)	248.0 (199.7–334.2)	0.81
CRP [mg/l]	3.17 (1.75–5.60)	8.07 (3.17–46.8)	0.003
IL-6 [pg/mL]	1.60 (1.50–3.63)	4.79 (1.50–15.1)	0.07
Ferritin [µg/L]	158.0 (98.5–254.5)	351.0 (181.2–697.2)	0.002
Iron [µmol/L]	17.4 (12.0–22.0)	14.3 (8.42–21.4)	0.16
ALT [IU/L]	25.0 (15.2–50.2)	43.0 (33.2–64.5)	0.01
AST [IU/L]	22.0 (15.7–27.7)	37.0 (24.5–49.5)	0.001
Bilirubin [µmol/L]	6.24 (4.33–6.93)	6.13 (4.44–8.34)	0.89
ALP [IU/L]	57.0 (50.7–73.5)	82.0 (64.5–115.2)	<0.001
INR	0.97 (0.92–0.99)	0.95 (0.92–1.02)	0.72
Albumin [g/L]	40.0 (37.0–44.2)	36.5 (33.0–42.0)	0.01
HOMA-IR	2.12 (1.90–2.60)	3.51 (2.45–4.55)	0.009
Saturation %	97 (96–98)	96 (93–98)	0.02

Abbreviations: ALT—alanine transaminase, AST—aspartate transaminase, BMI—body mass index, CRP—C-reactive protein, GGT—gamma-glutamyl-transferase, HCT—hematocrit, HDL—high-density lipoprotein, HGB—hemoglobin, INR—international normalized ratio, LDL—low-density lipoprotein, WBC—white blood cells, PLT—platelet count, RBC—red blood cells.

**Table 5:** Comparison of biochemical parameters analyzed in the blood of COVID-19 patients with and without pneumonia.

Parameters	Patients with Pneumonia (*n* = 23)	Patients without Pneumonia (*n* = 47)	*p*
FGF-21 [pg/mL]	278.8 (132.0–485.7)	190.0 (84.5–557.9)	0.54
PTX3 [pg/mL]	2824.4 (2165.6–4035.2)	2278.2 (1816.4–3103.5)	0.05
Fetuin-A [µg/mL]	217.4 (188.2–248.7)	256.3 (218.8–285.6)	0.008
Irisin [ng/mL]	5.31 (3.13–7.81)	5.47 (4.01–6.37)	0.77
WBC [10^3^/µL]	6.18 (4.76–6.62)	5.70 (4.48–6.36)	0.31
HGB [mg/dL]	12.6 (11.9–13.7)	12.8 (12.3–13.2)	0.66
PLT [10^3^/µL]	301.0 (218.5–405.0)	232.0 (189.7–301.7)	0.02
CRP [mg/L]	6.32 (4.39–40.0)	3.75 (1.77–15.0)	0.04
IL-6 [pg/mL]	5.68 (1.50–17.4)	1.50 (1.50–3.69)	0.006
Ferritin [µg/L]	306.0 (197.0–654.0)	175.0 (91.0–315.0)	0.008
Iron [µmol/L]	17.5 (9.30–22.0)	16.4 (11.7–21.2)	0.79
ALT [IU/L]	31.0 (17.7–45.2)	37.0 (20.7–58.2)	0.33
AST [IU/L]	24.0 (17.5–42.0)	25.5 (18.5–45.5)	0.69
GGT [IU/L]	64.0 (39.0–185.2)	33.0 (20.0–60.0)	0.009
ALP [IU/L]	70.5 (55.0–114.5)	64.0 (54.0–77.0)	0.12
Bilirubin [µmol/L]	6.51 (4.64–8.55)	6.13 (4.45–7.50)	0.51
INR	1.01 (0.95–1.07)	0.95 (0.92–0.99)	0.03
Albumin [g/L]	34.5 (32.0–41.0)	40.0 (37.0–43.7)	0.004
HOMA-IR	2.35 (1.55–4.51)	2.42 (1.85–2.71)	0.42
Saturation %	94.5 (89–96)	97 (96–98)	0.00005

Abbreviations: ALT—alanine transaminase, AST—aspartate transaminase, BMI—body mass index, CRP—C-reactive protein, GGT—gamma-glutamyl-transferase, HCT—hematocrit, HDL—high-density lipoprotein, HGB—hemoglobin, INR—international normalized ratio, LDL—low-density lipoprotein, WBC—white blood cells, PLT—platelet count, RBC—red blood cells.

**Table 6:** Comparison of biochemical parameters analyzed in the blood of COVID-19 patients requiring and nonrequiring ICU care.

Parameters	ICU (*n* = 9)	Non-ICU (*n* = 61)	*p*
FGF-21 [pg/mL]	408.6 (307.7–482.9)	216.6 (93.4–487.0)	0.30
PTX3 [pg/mL]	4768.9 (2896.8–8394.5)	2278.2 (1876.8–3106.2)	<0.001
Fetuin-A [µg/mL]	193.9 (124.8–229.8)	252.3 (209.3–276.5)	0.01
Irisin [ng/mL]	3.92 (3.04–6.99)	5.59 (3.96–6.60)	0.27
WBC [10^3^/µL]	8.53 (6.24–10.9)	5.70 (4.42–6.42)	0.003
HGB [mg/dL]	11.3 (10.1–12.6)	12.8 (12.3–13.5)	0.01
PLT [10^3^/µL]	403.0 (273.7–461.2)	232.0 (189.2–308.0)	0.009
CRP [mg/L]	18.2 (5.81–101.0)	4.39 (1.98–11.9)	0.009
IL-6 [pg/mL]	13.5 (6.46–20.9)	1.60 (1.50–5.35)	0.001
Ferritin [µg/L]	765.5 (526.0–1235.0)	208.0 (110.5–381.7)	0.005
Iron [µmol/L]	14.3 (7.55–18.8)	17.4 (11.5–21.8)	0.42
ALT [IU/L]	43.0 (13.2–46.0)	34.0 (20.7–54.2)	0.75
AST [IU/L]	31.0 (19.2–47.2)	25.0 (17.0–43.0)	0.56
GGT [IU/L]	253.0 (103.2–376.5)	33.0 (20.0–62.0)	0.001
ALP [IU/L]	92.0 (58.5–140.2)	64.0 (54.0–80.2)	0.10
Bilirubin [µmol/L]	6.70 (4.89–11.5)	6.14 (4.59–7.77)	0.46
INR	1.03 (0.96–1.08)	0.95 (0.92–1.00)	0.09
Albumin [g/L]	33.0 (27.5–34.7)	40.0 (36.0–43.7)	0.005
HOMA-IR	4.48 (2.93–4.73)	2.16 (1.83–2.66)	0.03
Saturation %	92 (80–95,25)	97 (96–98)	0.001

Abbreviations: ALT—alanine transaminase, AST—aspartate transaminase, BMI—body mass index, CRP—C-reactive protein, GGT—gamma-glutamyl-transferase, HCT—hematocrit, HDL—high-density lipoprotein, HGB—hemoglobin, INR—international normalized ratio, LDL—low-density lipoprotein, WBC—white blood cells, PLT—platelet count, RBC—red blood cells.

**Table 7:** Comparison of biochemical parameters analyzed in the blood of COVID-19 patients with different ferritin levels.

Parameters	Ferritin ≤250 µg/L (*n* = 36)	Ferritin >250 µg/L (*n* = 28)	*p*
FGF-21 [pg/mL]	133.3 (74.4–316.4)	375.0 (137.2–589.7)	0.01
PTX3 [pg/mL]	2246.3 (1699.8–2862.6)	2981.2 (2214.5–5079.4)	0.001
Fetuin-A [µg/mL]	257.7 (238.3–311.1)	220.5 (178.5–264.1)	0.001
Irisin [ng/mL]	5.47 (4.19–5.78)	5.23 (3.34–7.89)	0.92
WBC [10^3^/µL]	5.70 (4.48–6.68)	5.73 (4.30–6.03)	0.27
HGB [mg/dL]	12.7 (12.3–13.4)	12.9 (12.4–13.0)	0.95
PLT [10^3^/µL]	253.0 (214.0–323.7)	231.0 (186.2–360.0)	0.43
CRP [mg/L]	2.63 (1.35–5.60)	6.04 (3.45–36.0)	0.001
IL-6 [pg/mL]	1.60 (1.50–3.91)	5.62 (1.50–16.7)	0.01
Iron [µmol/L]	17.85 (11.9–21.7)	14.3 (6.05–22.5)	0.20
ALT [IU/L]	27.0 (16.2–50.0)	44.5 (31.0–66.0)	0.02
AST [IU/L]	20.5 (15.5–28.5)	36.0 (25.0–51.0)	<0.001
GGT [IU/L]	26.0 (15.0–47.2)	62.5 (32.0–117.0)	0.001
ALP [IU/L]	64.0 (54.7–79.0)	64.0 (55.0–88.0)	0.89
Bilirubin [µmol/L]	6.60 (4.48–8.56)	5.99 (4.77–7.21)	0.59
INR	0.96 (0.92–0.99)	0.96 (0.92–1.05)	0.46
Albumin [g/L]	41.7 (38.5–44.2)	36.0 (32.5–41.0)	0.001
HOMA-IR	1.94 (1.15–2.37)	2.82 (2.45–3.73)	<0.001
Saturation %	97 (96–98)	96 (93–98)	0.03

Abbreviations: ALT—alanine transaminase, AST—aspartate transaminase, BMI—body mass index, CRP—C-reactive protein, GGT—gamma-glutamyl-transferase, HCT—hematocrit, HDL—high-density lipoprotein, HGB—hemoglobin, INR—international normalized ratio, LDL—low-density lipoprotein, WBC—white blood cells, PLT—platelet count, RBC—red blood cells.

**Table 8:** Comparison of biochemical parameters analyzed in the blood of COVID-19 patients with and without iron deficiency.

Parameters	Iron ≤ 16.8 µmol/L (*n* = 32)	Iron > 16.8 µmol/L (*n* = 32)	*p*
FGF-21 [pg/mL]	355.0 (128.0–659.6)	163.9 (94.1–366.0)	0.09
PTX3 [pg/mL]	2910.7 (2268.3–3683.3)	2072.3 (1681.6–2324.5)	<0.001
Fetuin-A [µg/mL]	232.1 (179.3–265.6)	256.4 (220.5–292.2)	0.04
Irisin [ng/mL]	4.05 (3.14–5.70)	5.80 (4.76–7.83)	0.002
WBC [10^3^/µL]	5.48 (4.76–6.06)	6.01 (4.30–6.68)	0.62
HGB [mg/dL]	12.7 (12.2–13.1)	12.8 (12.4–13.9)	0.31
PLT [10^3^/µL]	227.0 (202.2–265.2)	290.0 (203.5–347.7)	0.05
CRP [mg/L]	12.6 (4.21–38.7)	2.77 (1.21–5.13)	<0.001
IL-6 [pg/mL]	5.42 (2.62–16.7)	1.50 (1.50–3.60)	<0.001
Ferritin [µg/L]	267.0 (111.0–526.0)	175.0 (110.7–258.2)	0.15
ALT [IU/L]	37.0 (20.5–56.7)	28.0 (19.2–48.0)	0.44
AST [IU/L]	25.5 (21.0–41.0)	23.0 (16.2–44.7)	0.24
GGT [IU/L]	50.0 (30.5–175.7)	30.0 (18.7–67.2)	0.05
ALP [IU/L]	76.0 (56.0–103.2)	61.0 (54.0–72.0)	0.02
Bilirubin [µmol/L]	6.00 (4.48–8.97)	6.32 (4.57–7.86)	0.74
INR	0.98 (0.91–1.02)	0.95 (0.93–0.97)	0.78
Albumin [g/L]	38.0 (33.2–42.7)	41.0 (35.0–44.0)	0.14
HOMA-IR	2.67 (1.68–4.25)	2.16 (1.90–2.58)	0.13
Saturation %	97 (95.5–98)	97 (95.75–98)	0.80

Abbreviations: ALT—alanine transaminase, AST—aspartate transaminase, BMI—body mass index, CRP—C-reactive protein, GGT—gamma-glutamyl-transferase, HCT—hematocrit, HDL—high-density lipoprotein, HGB—hemoglobin, INR—international normalized ratio, LDL—low-density lipoprotein, WBC—white blood cells, PLT—platelet count, RBC—red blood cells.

**Table 9:** Correlations between hepatokines concentration and biochemical parameters analyzed in the blood of COVID-19 patients.

	FGF-21		PTX-3		Fetuin-A		Irisin	
	r	*p*	r	*p*	r	*p*	r	*p*
FGF-21	-	-	0.32	0.008	−0.28	0.02	−0.08	0.48
PTX-3	0.32	0.008	-	-	−0.46	<0.001	−0.28	0.02
Fetuin-A	−0.28	0.02	−0.46	<0.001	-	-	0.44	<0.001
Irisin	−0.08	0.48	−0.28	0.02	0.44	<0.001	-	-
WBC	0.04	0.74	−0.21	0.09	0.03	0.76	0.13	0.32
HGB	0.05	0.67	−0.06	0.36	0.24	0.06	0.17	0.18
PLT	−0.11	0.36	−0.25	0.04	0.13	0.28	0.31	0.01
CRP	0.22	0.10	0.52	<0.001	−0.45	<0.001	−0.41	0.002
IL-6	0.48	<0.001	0.48	<0.001	−0.37	0.005	−0.23	0.08
Ferritin	0.25	0.04	0.38	0.002	−0.44	<0.001	−0.06	0.64
Iron	−0.16	0.21	−0.48	<0.001	0.26	0.03	0.47	<0.001
ALT	0.01	0.98	−0.002	0.98	−0.06	0.60	0.05	0.64
AST	0.17	0.17	0.20	0.11	−0.16	0.18	−0.08	0.53
GGT	0.20	0.15	0.13	0.34	−0.31	0.02	0.15	0.28
ALP	−0.10	0.42	−0.05	0.65	0.07	0.56	0.03	0.77
Bilirubin	−0.004	0.97	−0.21	0.10	−0.02	0.87	0.02	0.85
INR	−0.42	0.05	0.03	0.88	−0.30	0.15	−0.41	0.04
Albumin	−0.16	0.19	−0.46	<0.001	0.62	<0.001	0.24	0.05

Abbreviations: ALT—alanine transaminase, AST—aspartate transaminase, BMI—body mass index, CRP—C-reactive protein, GGT—gamma-glutamyl-transferase, HCT—hematocrit, HDL—high-density lipoprotein, HGB—hemoglobin, INR—international normalized ratio, LDL—low-density lipoprotein, WBC—white blood cells, PLT—platelet count, RBC—red blood cells.

## Data Availability

Derived data supporting the findings of this study are available from the corresponding author (D.S.) on request.

## Supplementary Materials

The following are available online at https://www.mdpi.com/article/10.3390/biom11101422/s1, Supplementary Table S1: Comparison of biochemical parameters analyzed in the blood of male and female COVID-19 patients, Supplementary Table S2: Comparison of biochemical parameters analyzed in the blood of COVID-19 patients presenting different infection symptoms, Supplementary Table S3: Comparison of biochemical parameters analyzed in the blood of COVID-19 patients according to ALT activity.
