# Supplementary material for: Fetuin-A Deficiency but Not Pentraxin 3, FGF-21, or Irisin, Predisposes to More Serious COVID-19 Course
Source: Biomolecules. 2021 Sep 28;11(10):1422. doi: 10.3390/biom11101422 (PMC8533535; doi:10.3390/biom11101422)
Supplement: Supplementary file 1 [file biomolecules-11-01422-s001.zip › biomolecules-1359256-supplementary.pdf]

Supplementary Table 1. Comparison of biochemical parameters analyzed in the blood of male and female COVID-19 patients.

Parameters	Females (n = 43)	Males (n = 27)	p
FGF-21 [pg/ml]	279.8 (100.6-536.9)	249.1 (83.0-410.6)	0.26
PTX3 [pg/ml]	2304.6 (1869.8-3212.1)	2740.1 (2194.8-3157.0)	0.21
Fetuin-A [μg/ml]	243.5 (193.9-275.7)	246.6 (217.4-270.8)	0.67
Irisin [ng/ml]	5.61 (4.47-6.51)	4.12 (3.73-6.74)	0.15
WBC [10^3/μl]	5.66 (4.42-6.68)	5.99 (4.90-6.21)	0.95
HGB [mg/dl]	12.7 (12.2-13.2)	12.8 (12.5-14.0)	0.28
PLT [10^3/μl]	250.5 (205.0-329.0)	238.5 (186.0-336.5)	0.70
CRP [mg/l]	3.90 (1.66-10.7)	6.24 (2.98-23.9)	0.10
IL-6 [pg/ml]	2.51 (1.50-6.16)	3.53 (1.50-6.62)	0.98
Ferritin [μg/l]	176.0 (109.5-351.0)	307.0 (179.0-665.5)	0.04
Iron [μmol/l]	17.4 (11.7-22.5)	14.3 (10.1-19.7)	0.29
ALT [IU/l]	42.5 (26.0-61.5)	25.0 (12.5-41.5)	0.01
AST [IU/l]	30.0 (18.5-45.5)	23.0 (17.2-29.5)	0.16
GGT [IU/l]	34.0 (20.5-68.5)	35.0 (23.7-94.5)	0.51
ALP [IU/l]	64.0 (54.0-75.7)	61.0 (53.5-90.0)	0.65
Bilirubin [μmol/l]	6.87 (4.70-8.56)	5.97 (4.49-6.67)	0.07
INR	0.95 (0.92-0.98)	1.01 (0.96-1.07)	0.06
Albumin [g/l]	40.0 (36.0-43.0)	40.0 (34.0-43.0)	0.44
HOMA-IR	2.09 (1.76-2.68)	2.52 (1.93-4.40)	0.13
Saturation %	97 (96-98)	96 (92.75-98)	0.20

Abbreviations: ALT - alanine transaminase, AST - aspartate transaminase, BMI - body mass index, CRP - C-reactive protein, GGT - gamma-glutamyl-transferase, HCT - hematocrit, HDL - high-density lipoprotein, HGB - hemoglobin, INR - international normalized ratio, LDL - low-density lipoprotein, WBC - white blood cells, PLT - platelet count, RBC - red blood cells.

Supplementary Table 2. Comparison of biochemical parameters analyzed in the blood of COVID-19 patients presenting different infection symptoms.

Parameters	Patients with cough and dyspnea (n = 41)	Patients with isolated cough (n = 20)	p
FGF-21 [pg/ml]	278.8 (156.1-414.7)	192.9 (91.5-594.9)	0.69
PTX3 [pg/ml]	2557.6 (2101.3-3045.9)	2179.4 (1866.4-2812.7)	0.24
Fetuin-A [µg/ml]	231.2 (211.1-262.9)	238.8 (187.4-260.2)	0.71
Irisin [ng/ml]	5.80 (3.93-7.97)	5.61 (4.33-6.41)	0.34
WBC [10^3/µl]	5.99 (5.11-6.66)	5.93 (4.86-6.97)	1.00
HGB [mg/dl]	13.1 (12.6-13.9)	12.5 (12.0-13.0)	0.03
PLT [10^3/µl]	279.0 (192.5-330.5)	284.5 (206.5-368.0)	0.67
CRP [mg/l]	5.88 (2.43-11.9)	5.06 (1.86-14.6)	0.88
IL-6 [pg/ml]	3.04 (1.50-11.5)	2.03 (1.50-15.1)	1.00
Ferritin [µg/l]	285.0 (89.3-526.0)	202.0 (141.0-525.0)	0.95
Iron [µmol/l]	15.9 (10.1-21.7)	17.4 (6.77-23.3)	0.57
ALT [IU/l]	25.0 (15.0-37.0)	45.0 (32.5-75.5)	0.003
AST [IU/l]	23.0 (17.2-36.0)	30.0 (20.2-56.2)	0.18
GGT [IU/l]	62.0 (24.0-73.0)	33.5 (18.0-59.0)	0.13
ALP [IU/l]	61.0 (51.5-57.7)	64.0 (54.5-75.5)	0.72
Bilirubin [µmol/l]	6.02 (4.57-7.86)	6.16 (4.70-8.20)	0.98
INR	0.99 (0.95-1.06)	0.93 (0.92-0.95)	0.13
Albumin [g/l]	38.0 (34.0-43.0)	41.0 (37.2-42.0)	0.50
HOMA-IR	2.31 (1.13-3.21)	2.04 (1.76-2.71)	1.00
Saturation %	96 (90-98.75)	97 (94.5-98)	0.84

Abbreviations: ALT - alanine transaminase, AST - aspartate transaminase, BMI - body mass index, CRP - C-reactive protein, GGT - gamma-glutamyl-transferase, HCT - hematocrit, HDL - high-density lipoprotein, HGB - hemoglobin, INR - international normalized ratio, LDL - low-density lipoprotein, WBC - white blood cells, PLT - platelet count, RBC - red blood cells.

Supplementary Table 3. Comparison of biochemical parameters analyzed in the blood of COVID-19 patients according to ALT activity.

**Parameters**	**ALT activity < 40 IU/L (n = 36)**	**ALT activity ≥ 40 IU/L (n = 34)**	**p**
FGF-21 [pg/ml]	277.7 (96.3-529.9)	240.2 (103.3-507.0)	0.82
PTX3 pg/ml]	2357.5 (1981.3-3080.4)	2254.0 (1723.8-3491.3)	0.63
Fetuin-A [µg/ml]	240.0 (202.9-270.6)	265.6 (205.6-321.9)	0.48
Irisin [ng/ml]	5.19 (3.94-5.85)	5.61 (3.77-7.62)	0.40
WBC [10^3/µl]	5.88 (4.74-6.34)	5.76 (4.40-6.84)	0.89
HGB [mg/dl]	12.8 (12.3-13.8)	12.6 (11.9-13.0)	0.20
PLT [10^3/µl]	232.0 (189.2-307.7)	260.0 (202.7-342.2)	0.42
CRP [mg/l]	4.79 (1.93-8.18)	3.90 (2.06-25.2)	0.77
IL-6 [pg/ml]	3.35 (1.50-5.87)	1.51 (1.50-7.45)	0.69
Ferritin [µg/l]	176.0 (97.7-322.0)	262.0 (133.2-595.5)	0.12
Iron [µmol/l]	16.5 (11.1-21.7)	16.5 (11.2-22.3)	0.99
AST [IU/l]	18.0 (15.0-23.0)	41.5 (20.0-51.0)	< 0.001
GGT [IU/l]	26.5 (15.0-49.0)	59.0 (32.2-108.2)	0.003
Bilirubin [µmol/l]	5.88 (4.33-6.93)	6.71 (5.02-8.48)	0.03
ALP [IU/l]	56.0 (50.0-69.0)	66.0 (59.5-89.5)	0.002
INR	0.99 (0.92-1.03)	0.95 (0.92-0.97)	0.24
Albumin [g/l]	40.0 (35.0-43.0)	38.5 (34.0-42.0)	0.41
HOMA-IR	2.03 (1.48-2.54)	2.67 (2.04-3.68)	0.02
Saturation %	97 (96-98)	97 (94.75-98)	0.17

Abbreviations: ALT - alanine transaminase, AST - aspartate transaminase, BMI - body mass index, CRP - C-reactive protein, GGT - gamma-glutamyl-transferase, HCT - hematocrit, HDL - high-density lipoprotein, HGB - hemoglobin, INR - international normalized ratio, LDL - low-density lipoprotein, WBC - white blood cells, PLT - platelet count, RBC - red blood cells.
